# Small RNA-mediated responses to low- and high-temperature stresses in cotton

**DOI:** 10.1038/srep35558

**Published:** 2016-10-18

**Authors:** Qiongshan Wang, Nian Liu, Xiyan Yang, Lili Tu, Xianlong Zhang

**Affiliations:** 1 National Key Laboratory of Crop Genetic Improvement, Huazhong Agricultural University, Wuhan, Hubei, 430070, China

## Abstract

MicroRNAs (miRNAs) are one class of endogenous non-coding RNAs modulating the expression of target genes involved in plant development and stress tolerance, by degrading mRNA or repressing translation. In this study, small RNA and mRNA degradome sequencing were used to identify low- and high-temperature stress-responsive miRNAs and their targets in cotton (*Gossypium hirsutum*). Cotton seedlings were treated under different temperature conditions (4, 12, 25, 35, and 42 °C) and then the effects were investigated. In total, 319 known miRNAs and 800 novel miRNAs were identified, and 168 miRNAs were differentially expressed between different treatments. The targets of these miRNAs were further analysed by degradome sequencing. Based on studies from Gene Ontology and Kyoto Encyclopedia of Genes and Genomes, the majority of the miRNAs are from genes that are likely involved in response to hormone stimulus, oxidation-reduction reaction, photosynthesis, plant–pathogen interaction and plant hormone signal transduction pathways. This study provides new insight into the molecular mechanisms of plant response to extreme temperature stresses, and especially the roles of miRNAs under extreme temperatures.

Temperature is an important factor limiting the geographical distribution and growing season of plants, and plants often suffer severe climatic damages such as causing tissue injury and delayed growth. Most organisms undergo optimal growth within a narrow temperature range, and can only tolerate minor fluctuations^1^. However, fluctuations beyond a threshold lead to low- or high-temperature stresses. Plants evolved complex mechanisms to cope with unfavourable conditions during the long-term evolutionary process^2^. Abiotic stresses affect plant development and production through effects on gene expression patterns under the control of different regulators such as non-coding RNAs and transcription factors^3^,^4^.

Small RNAs (sRNAs) have emerged as key post-transcriptional gene regulatory molecules in plants and animals. There are two classes of endogenous sRNAs: small interfering RNAs (siRNAs) and microRNAs (miRNAs) in plants. miRNAs are endogenous sRNAs having transcriptional and post-transcriptional regulatory effects on plant development and adaptive responses to stresses^5^. miRNAs predominantly play important gene-regulatory roles through base-pairing with target mRNAs for cleavage or translational repression^6^. They play regulatory roles in almost all aspects of plant development and response to stress^7^,^8^, and have been shown to be either up- or down-regulated in plant responses to abiotic stresses such as cold, salt, heat, drought, and oxidative stress^9^,^10^.

Low- and high-temperature stresses are two of the most important environmental factors affecting plant growth and development, but the roles of miRNAs in the plant’s response are not well understood. Although some miRNAs responding to stress are deeply conserved among various plant species, species-specific miRNAs with roles in regulatory networks associated with stress tolerance may arise from adaptation to long-term growth in stressful environments^11^. Recently, three conserved miRNAs and 25 predicted miRNAs have been identified by deep sequencing in response to cold stress in *Brachypodium*^12^. Several miRNAs respond to heat stress in wheat and barley, including miR160, miR166, miR167^13^,^14^.

Although temperature stress-responding miRNAs have been extensively investigated in several plant species, there has been no systematic research on miRNA profiles in cotton. Cotton is one of the most important commercial crops and is a major source of fiber for textiles in the world. Much focus has been directed to the mechanisms of cotton fiber development, quality and yield^15^. This includes analysis of miRNA expression during fiber development^16^, miRNA expression in biotic stress^17^ and abiotic stress responding to drought and salinity^18^. However, little work has been reported for cotton miRNAs in low- and high-temperature stresses, which can reduce fiber quality and yields.

The aim of this study was to profile the miRNA and their target mRNAs in cotton subjected to low- and high-temperature stresses, using high-throughput sequencing analysis and degradome sequencing. These findings reveal a putative miRNA-mediated regulatory network with a critical role in the response to low- and high-temperature stress in this important crop.

## Results

### Performance of seedlings under temperature stress conditions

**Figure 1:**
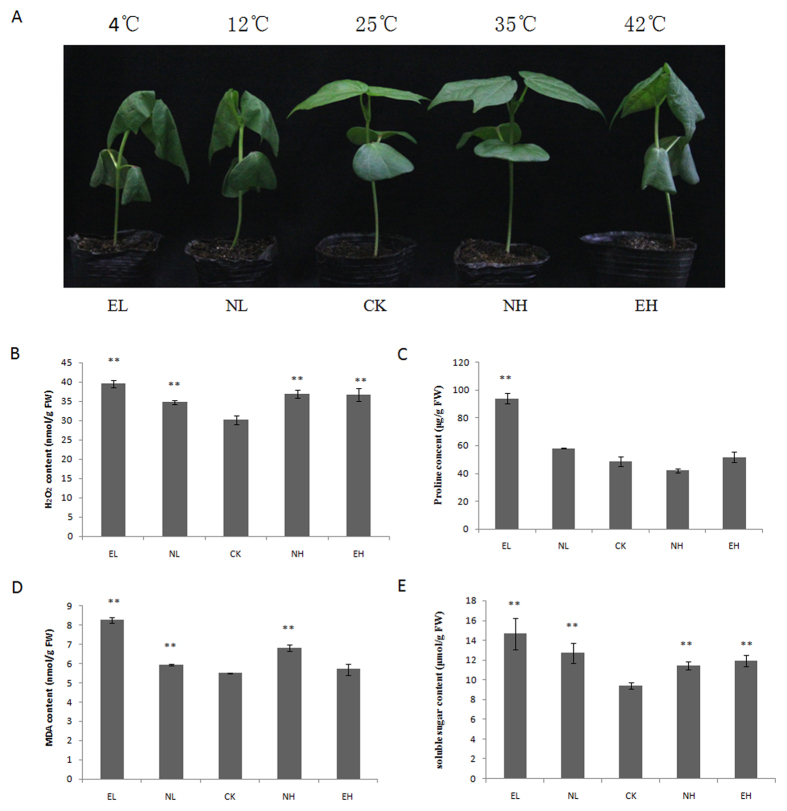
Response of two-leaf-stage cotton seedlings to temperature stress. **(A**) Performance of cotton seedlings under different temperature stresses for 8 h; (**B**) H_2_O_2_ contents of seedlings in EL, NL, CK, NH, EH; (**C**) Proline contents of seedlings in EL, NL, CK, NH, EH; (**D**) MDA contents of seedlings in EL, NL, CK, NH, EH; (**E**) Soluble sugar contents of seedlings in EL, NL, CK, NH, EH. Errorbars represent standard error. P-value was considered to be statistically significant with (*p < 0.05) or (**p < 0.01). EL, Extreme low temperature (4 °C); NL, Normal low temperature (12 °C); CK, Control (25 °C); NH, Normal high temperature (35 °C); EH, Extreme high temperature (42 °C).

To evaluate the biological and physiological performances during temperature treatments, we grew *G. hirsutum cv.*YZ1 seedlings under different temperature stress conditions (4, 12, 35, and 42 °C), each for 8 h, with the seedlings grown at 25 °C as control, all other environmental conditions being identical. Leaves of seedlings grown at 4 °C, 12 °C and 42 °C showed strong wilting, while seedlings grown at 25 °C and 35 °C showed no wilting (Fig. 1A). We measured four parameters of the leaves: H_2_O_2_ contents, proline contents, malondialdehyde (MDA) and soluble sugar contents. Overall, the contents of all physiological parameters were higher in treatments than in control especially under 4 °C conditions, except for MDA at 42 °C and proline at 12 °C, 35 °C and 42 °C (Fig. 1B–E).

### High-throughput sequencing of small RNAs in cotton

To characterize sRNA profiles in plants grown under the different temperature regimes, five sRNA libraries were constructed from total RNA isolated from leaves of plants treated for 8 h, with two sample replicates for each treatment. These libraries were sequenced by an Illumina Solexa Sequencer and a summary of the sequencing and data analysis strategy is shown in Fig. S1. The five libraries generated 11,804,407.5 clean reads at 4 °C (Extreme low temperature; EL); 11,848,760.5 clean reads at 12 °C (Normal low temperature; NL); 11,825,387 clean reads at 25 °C (Control; CK); 11,819,102 clean reads at 35 °C (Normal high temperature; NH); and 11,802,474 clean reads at 42 °C (Extreme high temperature; EH). On average, 85.97% (unique reads, Table 1) and 92.13% (total reads, Table 1) reads were successfully matched back to the AD genome of *G. hirsutum*, after removing poor quality reads and adapter sequences. For annotation purposes, the sRNAs were grouped into several classes (Table 1). Overall, the clean full-length reads that generated and matched were similar among the five libraries.

**Figure 2:**
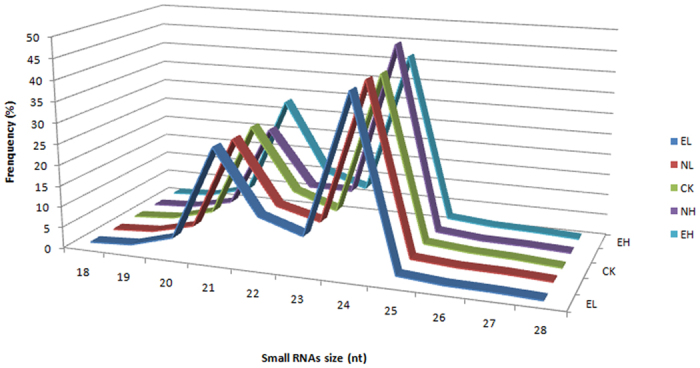
Length distribution of small RNA reads. EL, Extreme low temperature (4 °C); NL, Normal low temperature (12 °C); CK, Control (25 °C); NH, Normal high temperature (35 °C); EH, Extreme high temperature (42 °C).

The size distribution of all sequences with lengths between 18 and 28 nt was determined. A similar size distribution was observed for each of the five libraries, and a large proportion of the sRNAs were 20~24 nt long, in which the 24 nt read was the most abundant (41.6% to 46.6% of the five libraries), followed by 21 nt (Fig. 2). The sRNA abundance and size in cotton is largely consistent with the other plant species, such as *Arabidopsis thaliana*^19^, *Oryza sativa*^20^, *Glycine max*^21^, indicating that the majority of sRNAs in plants are 24 nt.

### Identification of known miRNAs

We identified known miRNAs by using homologous analysis to find sRNA sequences with criteria for selection of a length of at least 18 nt, and a maximum of two mismatches compared to all known plant miRNA sequences deposited in miRBase21.0 in the five small RNA libraries. After removing repeat sequences, 319 annotated known miRNAs belonging to 144 families were identified (Table S1); out of these, 313 were from the 4 °C treated plants, 313 from the 12 °C, 311 from the 25 °C, 311 from the 35 °C, and 312 from the 42 °C treated plants. The miRNAs had a very broad range of expression levels, from millions of sequence reads to fewer than 10. 44 miRNA families are represented by multiple members, from 2 to 20 (Fig. S2), such as the miR156 family (20 members) and the miR166 family (19 members); but 100 other miRNA families were represented by only one member. The most abundant miRNA families were found to be miR157, miR166, miR156 and miR167 (Table S2), whose expression levels were >10000 transcripts per million (TPM) clean tags, and these are highly conserved in mosses, eudicots and monocots^22^,^23^.

**Figure 3:**
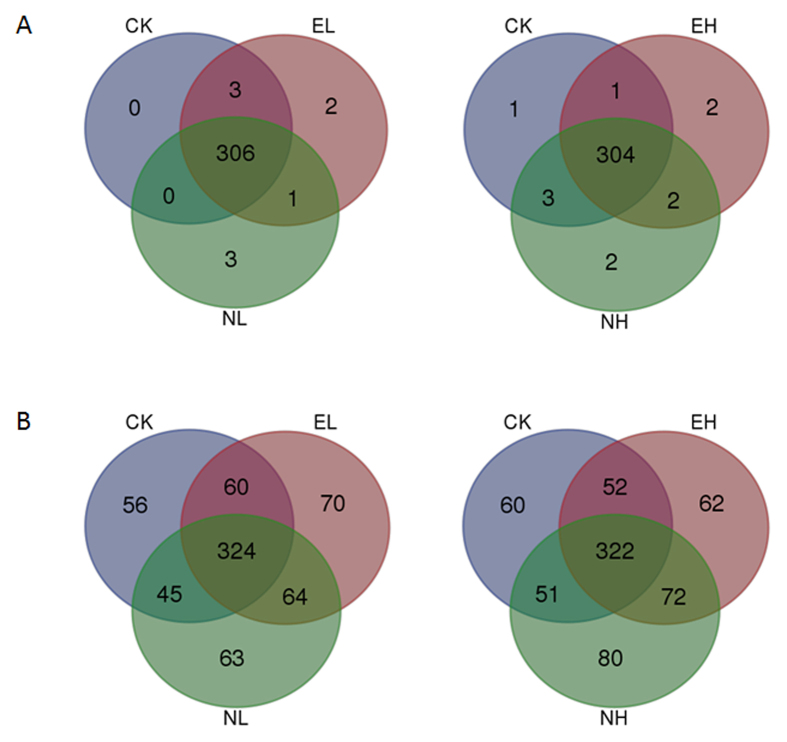
Distribution of miRNAs among treatments. **(A**) Known miRNAs in low-temperature treatments and control (left), known miRNAs in high-temperature treatments and control (right); (**B**) Novel miRNAs in low-temperature treatments and control (left); novel miRNAs in high-temperature treatments and control (right). EL, Extreme low temperature (4 °C); NL, Normal low temperature (12 °C); CK, Control (25 °C); NH, Normal high temperature (35 °C); EH, Extreme high temperature (4 °C).

More than 300 miRNAs were common across the five libraries, and among these known miRNAs less than 20 miRNAs were specific to temperature treatments. For example, gh-miR397-5p in EH (42 °C) and gh-miR8767c in NH (35 °C) were expressed only in high temperature-treatment samples, while gh-miR8779, gh-miR169h-3p and gh-miR7484c were expressed only in low temperature-treatment samples (4 °C, 12 °C). In addition, high temperature-treatment libraries shared 2 miRNA families that did not occur in the 25 °C library, gh-miR7484c and gh-miR171a-2. There was only one common miRNA, gh-miR399a-2, in the two low temperature treated libraries (Fig. 3A).

Of the 144 miRNA families, 21 nt long miRNAs represented the most abundant in size, which included 52.35% known miRNAs, followed by 24 nt sequences (22.26%) and 22 nt sequences (10.97%) in the known miRNA families (Table S3).

### Identification of potentially novel miRNAs

Among different RNA classifications, unannotated sRNAs accounted for the greatest proportion of the sRNAs. We selected the unknown sRNAs which could be mapped to the reference sequence to identify novel miRNAs by using Mireap software. The stem-loop structures matched to miRNA precursors were used to predict fold-back RNA secondary structure. The sequences localized inside the stem-loop structures in the 3′ or 5′ arms were regarded as mature miRNAs. We found 800 sequences supposed to be novel miRNAs with high confidence - on average 633 in the 4 °C samples, 617 in the 12 °C, 605 in the 25 °C, 630 in the 35 °C, and 613 in the 42 °C samples. Among these 800 novel miRNAs, about 350 were common across all five libraries, and about 60 miRNAs were in 4 °C, 12 °C, 35 °C and 42 °C samples (Fig. 3B). Analysis of nucleotide bias at each position in miRNAs showed that the first nucleotide of miRNAs tended to be uracil (U) (Fig. S3), which was conserved across many species^24^. Most of the novel miRNA in all five samples showed diversified expression levels. However, the abundance of these novel miRNAs was low, each generating fewer than 100 reads, which represents a lower expression level than that of known miRNAs. The length of these novel miRNAs ranged from 20 nt to 23 nt, in which 21 nt represented the most plentiful class, at more than 50%.

### miRNA expression in response to different temperatures

**Figure 4:**
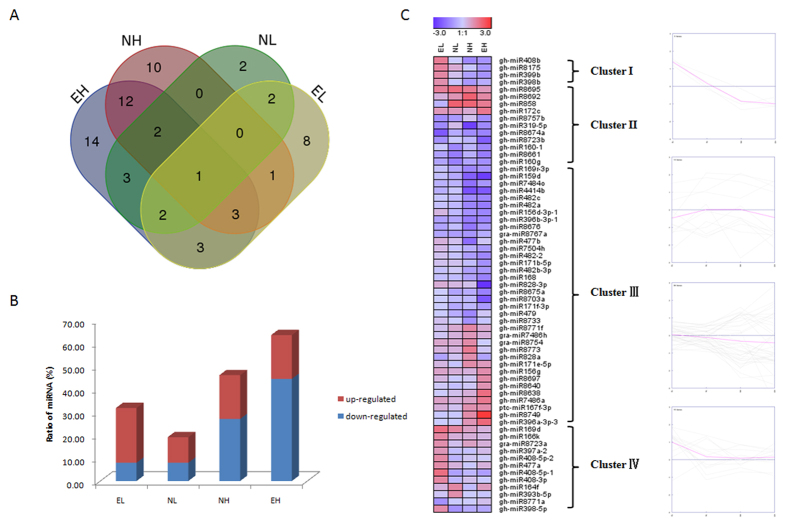
Comparison of known miRNAs identified between temperature stress and control libraries. **(A**) Venn diagram of temperature stress responsive miRNAs at EH, EL, NH and EH; (**B**) The overview of miRNAs highly responsive to temperature stress; (**C**) Heatmaps of differently expressed known miRNAs in EL, NL, NH and EH treatments, four miRNA clusters are shown on the right. Red, up-regulated; blue, down-regulated. EL, Extreme low temperature (4 °C); NL, Normal low temperature (12 °C); NH, Normal high temperature (35 °C); EH, Extreme high temperature (42 °C).

Among the identified miRNAs, 306 known miRNAs and 249 novel miRNAs, whose expression levels were more than 5 TPM, were used to analyse differential expression between temperature regimes. The results presented in Table S4 show that 63 out of 306 (20.6%) known miRNAs, and 105 out of 249 (42.2%) novel miRNAs, were expressed differentially in the five libraries (log2 Ratio ≥ 1 or ≤ −1). Among the 63 differently expressed known miRNAs, only 23.8% were in both low-temperature stress (4 °C and 12 °C) and high-temperature stress (35 °C and 42 °C). There was only one miRNA common across these four different temperature stresses, gh-miR8695, which was up-regulated in all conditions. Most miRNAs were expressed differentially in specific low- or high-temperature stress, with 57.1% especially highly expressed in high-temperature stress (Fig. 4A). Interestingly, more differentially expressed miRNAs were present in high-temperature stress, with relatively few differentially expressed at 4 °C. More miRNAs were significantly down-regulated in plants under high-temperature stress than in the 25 °C reference treatment, while more miRNAs were up-regulated in low-temperature stress than at 25 °C (Fig. 4B). The most significant expression difference was seen for gh-miR828-3p, which was 3.9-fold down-regulated at 42 °C.

Cluster analysis revealed that four clusters were constructed based on the expression patterns of 63 known miRNAs in response to different temperature regimes (Fig. 4C). Cluster I contains miRNAs with a converse expression pattern in low- and high-temperature stress; Cluster II shows the same expression pattern both in low- and high-temperature stress. Some specific significant expression changes were observed under high-temperature stress in Cluster III; and Cluster IV contains miRNAs which are only differentially expressed in low-temperature stress (Fig. S4).

Among the 105 differentially expressed novel miRNAs identified in the five libraries, a quarter of them had the opposite expression trend in low- and high-temperature stress, and most of the expression levels were simultaneously up-regulated or down-regulated in low-temperature stress or high-temperature stress (Fig. S5). The novel miRNAs identified here were more obviously differentially expressed under temperature stress, although their expression levels were lower than those of known miRNAs. Most miRNAs (known and novel) were down-regulated significantly under high-temperature stress (Fig. S6).

### Identification of known and novel miRNA targets through degradome sequencing

Plant miRNAs can bind almost perfectly to their target genes via typical complementarity matching, and regulate post-transcriptional process by transcript degradation or translational inhibition^25^. Accurate validation of miRNA targets is important to elucidate potential biological functions of miRNAs. In our study, degradome sequencing technology was used to detect the miRNA targets cleaved by the identified candidate miRNAs. We acquired approximately 1 × 10^8^ raw reads from each library through the degradome sequencing. After alignment with the AD genome of *G. hirsutum*, when a mismatch threshold ≤2 was applied, 10,305,697 clean tags from the 4 °C library were obtained, 9,791,298 from the 12 °C library, 10,9789,76 from the 25 °C library, 9,854,917 from the 35 °C library, and 12,549,112 from the 42 °C library (Table 2).

The target mRNAs were further investigated to gain insight into the regulatory function of the miRNAs. A total of 950 target transcripts for 133 known miRNAs, and 590 candidate targets for 253 novel miRNAs, were identified. For the identification of cleavage sites, degradome peaks could be classified into five classes (categories 0, 1, 2, 3 and 4). Among the identified targets, the targets with category 0 or 1 were evaluated as the most significant, and category 0 was the most abundant category of known miRNAs (Table 3). miRNAs were found to be able to target various numbers of genes with a range of 1 to 24, of which gh-miR828a targeted the highest number of genes, reaching 24 different genes (Table S5). A total of 216 target transcripts for 63 differentially expressed miRNAs, including 32 known and 31 novel miRNAs were identified in our degradome libraries (Table S5 and Table S6).

**Figure 5:**
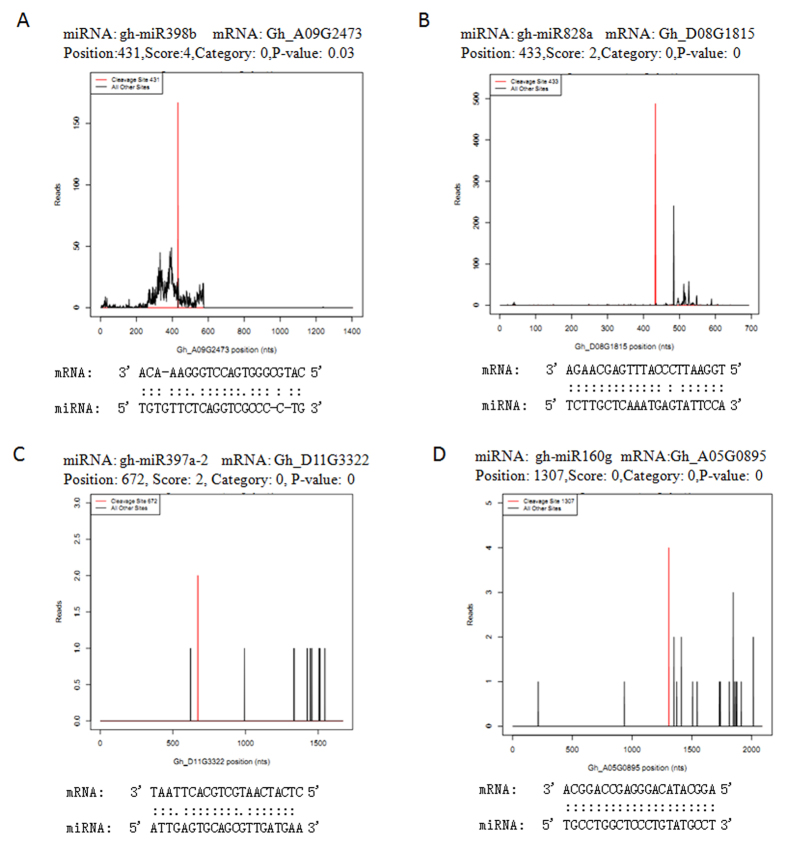
Cotton miRNA target alignment and its T-plot validated by degradome sequencing. The T-plots show the distribution of the degradome tags along the full length of the target mRNA sequence. Red lines indicate signatures consistent with miRNA-directed cleavage. (**A**) gh-miR398b and Gh_A09G2473 (chloroplast Cu/ZnSOD); (**B**) gh-miR828a and Gh_D08G1815 (MYB-like DNA-binding domain protein); (**C**) gh-miR397a-2 and Gh_D11G3322 (laccase1b); (**D**) gh-miR160g and Gh_A05G0895 (auxin response factor 3).

The results revealed that the potential target genes participated in various biological processes. Targets included genes encoded transcription factors, such as GRAS, MYB, NAC-domain, and ARF (auxin response factor), which were involved in the regulation of gene expression and signal transduction; others were disease resistance-related proteins of the CC-NBS-LRR class known as stress-responsive proteins; and other stress-induced proteins, such as laccase and heat shock proteins. In addition, mRNAs encoding proteins associated adaptive responses in plant growth and development, such as copper binding protein, transferases, phosphatases as well as other proteins of unclear functions, were identified as targets of miRNAs. Based on the degradome sequencing data, Fig. 5 shows examples of some confirmed stress-related miRNA targets as ‘target plots’ (T-plots).

### Functional enrichment for the miRNA target genes

In the present study, we conducted Gene Ontology (GO) and Kyoto Encyclopedia of Genes and Genomes (KEGG) pathway analysis for better understanding these differentially expressed miRNAs and their target genes. According to GO classifications, the ‘biological process’ category contained the largest number of target genes. These target genes predominantly participated in 53 biological process categories, 21 molecular function categories and 1 cellular component categories (Table S7). Most specific GO classification showed that the target genes were involved in biological processes associated with response to hormone stimulus (GO:0009725), single-organism transport (GO:0044765), defense response (GO:0006952), photosynthesis, and light harvesting (GO:0009765), regulation of transcription, and DNA-dependent (GO:0006355). Sequence-specific DNA binding (GO:0003700), hydrolase activity (GO:0016787), glycine dehydrogenase (decarboxylating) activity (GO:0004375), ATP citrate synthase activity (GO:0003878), and protein dimerization activity (GO:0046983) were significantly enriched among the most abundant classes in molecular function category. Nucleus (GO:0005634) was enriched in cellular component categories.

KEGG-based analysis allows linkage of 168 miRNAs and 216 targets to 26 pathways, and the significantly enriched pathways include photosynthesis-antenna proteins (ath00196), plant-pathogen interaction (ath04626), protein processing in endoplasmic reticulum (ath04141), porphyrin and chlorophyll metabolism (ath00860) (Table S8).

### Confirmation of predicted miRNAs by qRT–PCR

**Figure 6:**
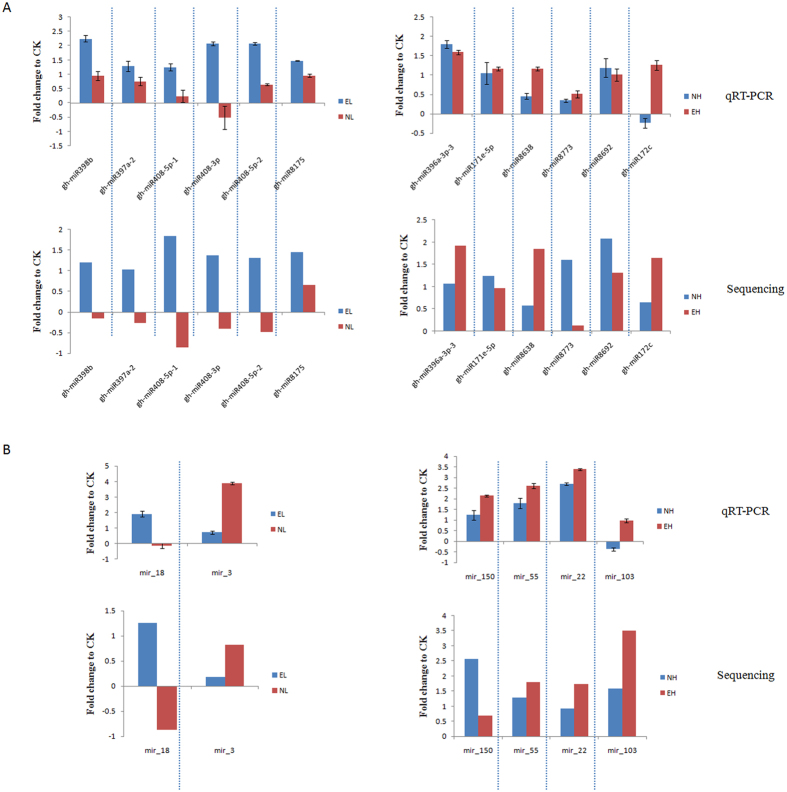
Fold-change of miRNA expression relative to CK (log2) by qRT-PCR. Expression was normalized by the level of *UBQ7* in qRT-PCR. All reactions of qRT-PCR were repeated three times for each sample and the results presented as the mean ± SD. (**A**) Known miRNA; (**B**) Novel miRNA. EL, Extreme low temperature (4 °C); NL, Normal low temperature (12 °C); CK, Control (25 °C); NH, Normal high temperature (35 °C); EH, Extreme high temperature (42 °C).

**Figure 7:**
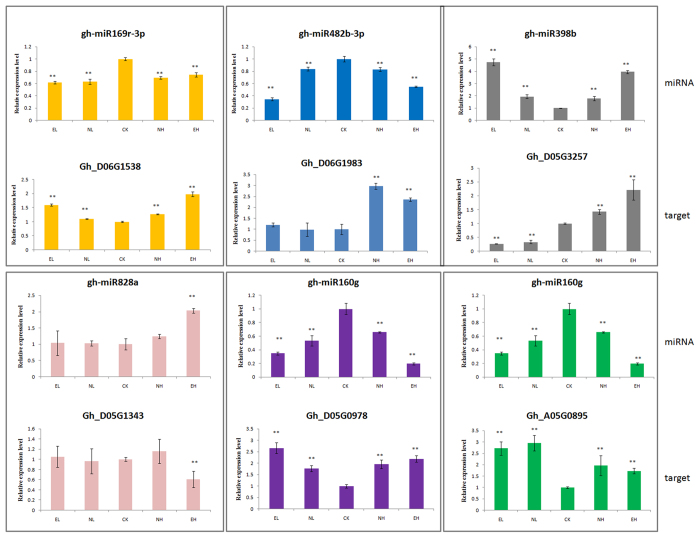
Expression profiles of miRNAs and their targets under temperature stress by qRT-PCR. All data were subjected to an analysis of variance (ANOVA) and the results presented as the mean ± SD. A P-value was considered to be statistically significant with (*p < 0.05) or (**p < 0.01). EL, Extreme low temperature (4 °C); NL, Normal low temperature (12 °C); CK, Control (25 °C); NH, Normal high temperature (35 °C); EH, Extreme high temperature (42 °C).

To validate the reliability of deep sequencing, we used qRT-PCR to analyse the expression of the miRNAs. Based on the high-throughput sequencing results, miRNAs were randomly selected for qRT–PCR (Fig. 6). Overall, expression patterns of these selected miRNAs obtained by qRT-PCR were consistent with the sequencing results. The miRNA expression trends are similar, indicating high reliability of the analysis. To validate whether the predicted target genes were really regulated by miRNAs under different temperature stresses, we conducted the expression analysis of the predicted target genes and their corresponding miRNAs, and the results revealed that the predicted target genes had an opposite expression profiles compared to the corresponding miRNAs (Fig. 7).

## Discussion

miRNAs, the post-transcriptional gene regulators, are known to play an important role in response to biotic and abiotic stresses. Environmental stresses may cause certain miRNAs differentially expressed or some novel miRNAs synthesized to cope with various stresses in plants. A few of stress-responsive miRNAs have been detected in plants in various biotic and abiotic stresses, including nutrient deficiency^26^, drought^27^, heat^13^, cold^28^, and bacterial infection stress^29^. There are also several abiotic stress-regulated miRNAs known in cotton, but most are associated with growth^30^,^31^, and response to drought and salinity stress^18^.

Most plants can regulate the physiological and biochemical processes by changing H_2_O_2_, MDA, proline and soluble sugar contents to adapt to temperature stresses in which ROS generation and membrane damage were induced^32^,^33^. In our study, the physiological parameters (H_2_O_2_, MDA, proline and soluble sugar contents) in response to temperature stresses were higher than control (Fig. 1), the results herein demonstrate that seedlings might be less capable of surviving from temperature stress conditions. Therefore, we used seedlings under different temperature stress conditions (4, 12, 35, and 42 °C) to analyse the regulation by miRNAs.

**Figure 8:**
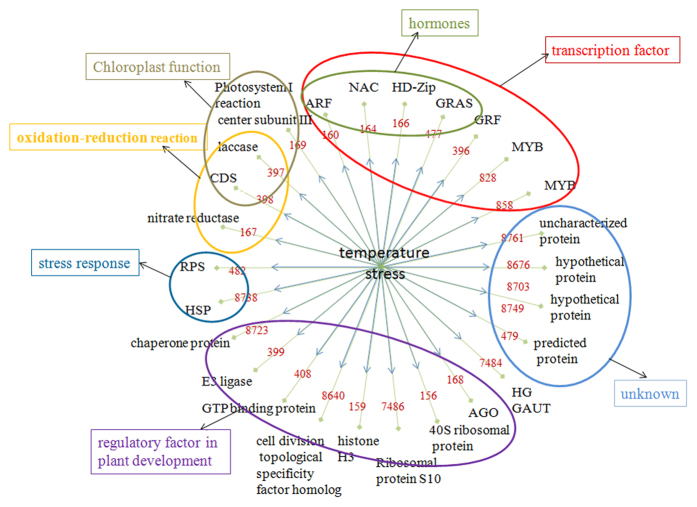
Summary of differentially expressed miRNAs and their targets genes involved in temperature stress. Numbers in internal circle with red color were miRNAs families. The genes in outer race represented the targets for counterparts in the line.

We identified 319 known miRNAs and 800 novel miRNAs in total (Table S1), and their corresponding precursors and prediction targets, expressed in response to temperature stress. Among all the miRNAs, 63 known miRNAs and 105 novel miRNAs were identified to be temperature stress-responding. Degradome sequencing led to the identification of 216 target genes for 32 known and 31 novel miRNAs (Table S5 and Table S6). 156 target genes for 32 known miRNAs represent members of 27 gene families, which were enriched in transcription factors; regulatory factors; stress-responsive proteins; proteins involved in oxidation-reduction reactions and photosynthesis, as well as other proteins whose functions are currently unclear (Fig. 8).

### miRNAs might regulate cotton growth homeostasis during temperature stress

Temperature stress negatively affects plant growth and development by causing tissue injury and delaying growth, and at least several of the stress-responding miRNAs can be expected to be involved in pathways that reprogramme metabolism and physiology. For example, temperature stress is known to disrupt the plant’s nutrient, metabolite and hormone homeostasis^34^,^35^.

Copper (Cu) is an essential micronutrient required for photosynthesis, oxidative responses, and other physiological processes^36^. There were several Cu homeostasis related miRNAs in low- and high-temperature stress, such as miR398 and miR397. miR398 is an important regulator of Cu homeostasis, and reduces the allocation of Cu to chloroplastic copper/zinc superoxide dismutases (Cu/Zn-SOD, CSDs) in response to low copper availability and makes Cu available for some essential processes in Arabidopsis^37^. The different expression patterns of miR398b detected under low- and high-temperature conditions reveal that miR398b may act as a mediator in response to temperature stress. In plants, lignin is the main component of secondary cell walls and its degradation is regulated by laccases, a kind of blue copper oxidase enzyme. The relative expression levels of the two carrot laccase-encoding genes *DcLac1* and *DcLac2* under heat and cold treatments are similar^38^. In cotton, our data predict that laccase is the target of gh-miR397a-2, and gh-miR397a-2 is significantly up-regulated at 4 °C compared with the 25 °C treatment. This suggests that nutrient and metabolite homeostasis is disturbed in low- and high-temperature stress, perhaps to mobilize resources to supporting plant growth and development during stress (Fig. S7).

Cu homeostasis and hormones had a complex reciprocal relationship in plants, and hormonal regulation requires matching special copper^39^. It was documented that any change in temperature would modify endogenous hormone levels^40^. ARF transcription factors regulate the expression of auxin-inducible genes by binding auxin-responsive promoters (ARPs)^41^. Previous studies revealed that some ARFs are targeted by miR160 and miR167^42^, which play a key role in attenuating plant growth and development under stress conditions^43^,^44^. Consistent with this, the miR160 family, including miR160-1 and gh-miR160g, are significantly down-regulated in both 12 °C and 42 °C treatments, and the targeted genes were identified as encoding ARF genes. Therefore gh-miR160g might participate in cotton seedling development and seedling tolerance to temperature stress through modulation of the auxin response. Furthermore, a novel miRNA mir-119, targeting an auxin-responsive protein, was found to decrease in high-temperature stress. This suggests that auxin signalling regulation in response to low- and high-temperature stress is worthy of further study.

Therefore, such a temperature shift induced a disruption of the plant’s overall homeostatic state including nutrient, metabolite and hormone homeostasis which may impact proteins encoding regulatory factors in plant development as shown in Fig. 8 to attenuate plant growth and metabolic rate in response to temperature stresses (Fig. S7).

### Chloroplast may be a sensor for temperature stress

The chloroplast is considered a sensor of environmental changes and plant stress responses^45^. In chloroplasts, the stabilization of the lamellar membrane systems through alterations in lipid composition is a key factor for tolerance, and damage to thylakoid membranes impairs photosynthesis and photosynthetic capacity^46^. Our results found significantly enriched GO terms in photosynthesis and light harvesting (GO:0009765) (Table S7), while KEGG-based pathway identifications were significantly enriched in photosynthesis-antenna proteins (ath00196) and porphyrin and chlorophyll metabolism (ath00860) (Table S8). gh-miR397a-2, which targets a chlorophyll a-b binding protein P4, was up-regulated in low-temperature stress, and gh-miR169r-3p, which targets Photosystem I reaction center subunit III (PasF), was down-regulated in high-temperature stress.

Superoxide radicals as the primary products in photo-reduction of dioxygen in chloroplasts are major consumers of Cu, which may cause lipid peroxidation and oxidative stress under stresses. Chloroplast is one of the major sites for the generation of ROS under stress, and causes damage to the photosynthetic apparatus and may function as a second messenger^47^. Some components in temperature stress have been found to function in protection from oxidative damage, and there were several genes related to ROS detected to be miRNA targets in cotton. Superoxide dismutases convert superoxide into molecular oxygen and hydrogen peroxidein plants. miR398 targets the mRNA of chloroplastic copper/zinc superoxide dismutase (CSD), a scavenger enzyme of ROS detoxifying superoxide radicals for degradation^48^,^49^. CSD2 is attached to the thylakoid of chloroplasts, where superoxide generated, so that it can localize and immediately sweep superoxide radicals. The decrease in expression level of miR398 led to increase in CSD expression which was important for plant tolerance to oxidative stress^50^. In Arabidopsis, transgenic plants expressing a form of the CSD2 showed higher ability to detoxify ROS superoxide radicals and regulation of the plant tolerance to oxidative stress conditions^51^. miR398 and the corresponding targets CSD genes are conserved across many plant species, and might have a ubiquitous role in oxidative stress management under various abiotic stresses. Our results show that gh-miR398b expression is up-regulated at 4 °C and down-regulated at 35 °C. The results suggested that miR398-regulated *GhCSD* genes play roles in temperature stress through protecting plants from oxidative damage. Interestingly, the mRNAs of a CSD gene targeted by a novel miRNA, mir-496, showed an up-regulated expression pattern at high-temperature stress. All of these results nevertheless suggest that temperature stress affects the chloroplasts and ROS, and the stress-responsive miRNAs are involved in associated pathways (Fig. S7), which is consistent with the results that H_2_O_2_, MDA, proline, and soluble sugar contents increased in response to temperature stresses.

### Transcription factors play a key role in the response to temperature stress

Transcription factors regulated by miRNAs may be crucial for plant tolerance to temperature stresses. Almost one third of the temperature-responsive miRNA-targeted transcription factors were shown to be related to regulation of plant growth and development. These include auxin-responsive genes, members of the YABBY2-like transcription factor family, the HD-Zip transcription factor family, the GRAS transcription factor family, the MYB-like family, the CIPK family, the eukaryotic translation initiation factor 2c family, and the zinc finger-like family (Fig. 8).

The *MYB* gene family is one of the largest families in plants, and some of its members are regulated by miRNAs. For instance, an R2R3-type MYB transcription factor, *MYB96*, is induced by cold stress in an ABA-independent manner and subsequently activates freezing tolerance^52^. Over-expression of *OsMYB55* in maize was found to improve plant growth and performance under high temperature and drought conditions^53^. In cotton, miR828 and miR858 regulate the homologous *MYB2* gene during both Arabidopsis trichome and cotton fibre development^54^. In the present study, we show that *GhMYB* is targeted by gh-miR828a and gh-miR858, and is up-regulated in high-temperature stress, suggesting a role for *GhMYB* targeting by miR828 and miR858 in response to high-temperature stress. In addition, miR166g and its target genes class III AtHD-ZIP regulates shoot apical meristem and lateral organ formation^55^. In our study, we found that the HD-Zip transcription factor family was also predicted to be a target mRNA of cotton miR166, and the expression level of gh-miR166k increased at 4 °C. Therefore, there may exist a regulatory interaction between miR166 targeted HD-ZIP and temperature stress.

### Abiotic and biotic stresses may somehow share common response pathway

Low- and high-temperature stresses were found to decrease the tolerance of plants to biotic stresses, so the genes encoding putative disease resistance proteins are regulated under stress conditions^56^,^57^. In the present study, miR482 family (gh-miR482-2, gh-miR482a, gh-miR482b-3p, gh-miR482c) was found to be down-regulated in high-temperature stress. Among the miR482 members, two of them targeted disease resistance proteins. gh-miR398b, which targeted a disease resistance protein RGA2, was differentially expressed both in low- and high-temperature stresses. These results indicated that stress inducible proteins were involved in temperature stresses (Fig. 8 and Fig. S7), so we propose that abiotic and biotic stresses may share some common regulatory mechanism, temperature stress may make the plant vulnerable to pathogens.

## Conclusions

In summary, we identified 63 known miRNAs and 105 novel miRNAs differentially expressed in response to low- and high-temperature stress. GO terms and KEGG-based enrichment analysis showed that most of the miRNAs involved in the response to temperature stress act by regulating genes associated with growth homeostasis, ROS, chloroplast function, plant–pathogen interaction and plant hormone signal transduction pathways. This provides a valuable platform for future functional analysis.

## Methods

### Plant culture and treatments

Upland cotton seeds (*Gossypium hirsutum cv.* YZ1) were grown in a growth chamber at 25 °C under a 16 hour/8 hour (light/dark) photoperiod. When the plants reached the two-leaf stage, they were treated under different temperature conditions (4, 12, 25, 35, and 42 °C) for 8 h. All leaves were harvested and immediately frozen in liquid nitrogen, and stored at −80 °C prior to RNA isolation. Three biological replicates were carried out for each treatment. We considered the samples in 25 °C without any treatment as the reference point for comparison.

### Total RNA extraction

Total RNA was extracted from leaf tissue using a modified guideline thiocyanate method^58^. Samples were ground in a mortar with liquid nitrogen, and RNA was extracted by ice-cold extraction buffer containing 1% β-mercaptoethanol. The supernatant was purified by phenol and chloroform and then precipitated by isopropanol and sodium acetate (3 M), washed with 75% ethanol. The RNA was air dried and dissolved in DEPC-treated water. All the RNA samples were quantified and equalised to ensure equal amounts of RNA from each treatment were available for library construction. The quality of RNA samples was evaluated using an Agilent 2100 Bioanalyzer (RIN ≥ 7.5; 28S:18S ≥ 1.3) (Agilent, Waldbronn, Germany).

### Construction of small RNA and degradome libraries

The sRNA libraries were constructed following a standard protocol (Illumina, USA). Small RNAs were purified from 10 μg of total RNA by polyacrylamide gel electrophoresis, and ligated first to a 5′ RNA adaptor and then to a 3′ RNA adaptor as previously described^28^. Purified RNAs were reverse-transcribed to cDNA, followed by PCR amplification to generate the DNA pool^16^. Five DNA pooled libraries were sequenced on an Illumina Solexa Sequencer at the Beijing Genomics Institute (BGI, http://www.genomics.cn/en/index) in Wuhan. Each sample was duplicated.

Five degradome libraries were constructed from cotton leaves to predict the potential target mRNAs following the methods described previously^59^. Briefly, a 5′ RNA adapter with a *Mme*I recognition site at the 3′ end was ligated to the resulting 42 bp fragments consisting of a free phosphate at the 5′ end, followed by reverse-transcribed to cDNA. After PCR amplification, they were digested by the enzyme *Mme*I, and ligated to Illumina 3′ TruSeq adaptors, followed by PCR amplification with library-specific index primers and a common 5′ primer for multiplex sequencing. Five DNA pools were sequenced on an Illumina Solexa Sequencer at BGI.

### Bioinformatics analysis of sequencing data

Raw sequences obtained from the five small RNA libraries and degradome libraries were first cleaned by filtering out low-quality tags, poly (A) tags, and tags with 3′ adaptor nulls, insert nulls, 5′ adaptor contaminants, or were smaller than 18 nt. Clean reads matching other small RNAs, including rRNA, snRNA, repeat RNA, tRNA, and snoRNA, were compared with *G. hirsutum* non-coding RNA sequences in the NCBI GenBank and Rfam databases. The remaining sequences from 18~24 nt long were used to BLAST against the miRBase 21.0 (http://www.mirbase.org/) to identify conserved miRNAs and novel 5p- and 3p-derived miRNAs. Only the sequences that were ≤2 mismatches with known miRNAs in miRBase were considered as conserved miRNAs, otherwise reads are defined as non-conserved reads. Unannotated reads were used for prediction of novel miRNAs according to the characteristic hairpin structure of microRNA precursors by the RNA-folding software Mireap (http://sourceforge.net/ projects/mireap/). We used the AD genome sequences of *G. hirsutum* as a miRNA positioning reference sequence to identify miRNA precursors^60^.

The 20~21 nt sequences of clean full-length reads collected from the degradome sequencing were used for subsequent analysis after removing low quality sequences and adapters. There were no mismatches allowed on the 10th and 11th nucleotides of mature miRNAs where the splice site on miRNA targets generally occurs in degradome analysis. Potential miRNA targets with a P-value of < 0.05 by PAREsnip software were retained, and T-plot figures were drawn. All target sequences were categorized into five classes based on the abundance of degradome tags indicating miRNA-mediated cleavage. The category 0~4 was determined as described^61^.

### Expression pattern of miRNAs between stress and control libraries

To investigate differentially expressed miRNAs between the control and treated libraries, the fold change of each identified miRNA was calculated as the ratio of read counts in the treatment libraries to the read counts in the control library followed by transformation of log2. The value of log2 Ratio ≥1 or ≤−1, indicating the ratio of RPKM values for the treatments and control libraries, were considered as significantly differentially expressed. To show differential expression profiles, heatmaps and clusters were constructed for the miRNAs using Genesis (http://genome.tugraz.at/genesisclient/genesisclient_description.shtml).

### qRT-PCR analysis

To further verify our identification results, chosen sequences were subjected to qRT-PCR. In the first step, 3 μg of total RNA was reverse-transcribed to cDNA for miRNAs using the Mir-X™ miRNA First-Strand Synthesis and SYBR Kit (Clontech, CA, USA). RNA was reverse-transcribed to cDNA for target genes using the SuperScript III reverse transcriptase (Invitrogen, Carlsbad, CA, USA). In the second step, quantitative real-time PCR (qRT-PCR) was carried out using the ABI Prism 7500 system (Applied Biosystems, Foster City, CA, USA) for 30 s at 95 °C, followed by 40 cycles of 5 s at 95 °C and 35 s at 60 °C. For qRT-PCR analysis, at least 5–10 plants of every line or treatment were sampled for each independent biological replicate^62^. The expression level of *UBQ7* was used as the internal control to standardize the RNA samples for each reaction. Three biological replicates for each experiment were performed. Error bars represent the ± SD. The Ct (2^−ΔΔCt^) was used to calculate the fold changes, and relative expression levels were shown as log2 fold changes. The primers used in the study are listed in Table S9.

### Measurement of physiological indexes

Endogenous H_2_O_2_ levels were detected by H_2_O_2_ Quantitative Assay Kit (Sangon Biotech, Shanghai, China). The contents of malondialdehyde (MDA) and soluble sugar and proline were measured according to the method described previously^63^,^64^.

### Function enrichment analysis

To evaluate the miRNA-gene regulatory network, target sequences were annotated using Blast2GO software for assigning GO terms to investigate putative functions. GO terms among a list of genes considered all genomic genes as the background. GO-controlled vocabularies describe three categories of biological process, molecular function and cellular component. The statistical significance of GO term enrichment was measured by Fisher’s exact test with a corrected FDR of <0.05. The main metabolic pathways associated linked to target genes can be predicted by KEGG enrichment analysis using KOBAS 2.0 (http://kobas.cbi.pku.edu.cn/home.do). KEGG enrichment analysis was obtained with a P-value of < 0.05.

## Additional Information

**How to cite this article**: Wang, Q. *et al*. Small RNA-mediated responses to low- and high-temperature stresses in cotton. *Sci. Rep.* **6**, 35558; doi: 10.1038/srep35558 (2016).

## Supplementary Material

Supplementary Figures

Supplementary Tables

## Figures and Tables

**Table 1:** The annotation and classification of small RNAs.

	Categories	EL		NL		CK		NH		EH	
		number	percent(%)	number	percent(%)	number	percent(%)	number	percent(%)	number	percent(%)
unique reads	exon_antisense	61364.5	1.40%	60250	1.36%	58039	1.37%	65286.5	1.33%	61891	1.44%
	exon_sense	127168.5	2.90%	148868	3.37%	138553.5	3.27%	129114	2.63%	124600	2.90%
	intron_antisense	111102	2.53%	109965	2.49%	107072.5	2.52%	123000.5	2.50%	108862.5	2.54%
	intron_sense	245072	5.58%	244110	5.52%	237775	5.61%	261570.5	5.33%	237855	5.54%
	rRNA	77406.5	1.76%	88122.5	1.99%	83260.5	1.96%	71711.5	1.46%	77391.5	1.80%
	repeat	14	0.00%	15	0.00%	17	0.00%	15	0.00%	14	0.00%
	snRNA	1452.5	0.03%	1646	0.04%	1411.5	0.03%	1398.5	0.03%	1444.5	0.03%
	snoRNA	630.5	0.01%	692	0.02%	615.5	0.01%	623.5	0.01%	617.5	0.01%
	tRNA	7394	0.17%	8141.5	0.18%	7033.5	0.17%	6189.5	0.13%	6547.5	0.15%
	miRNA	313	0.01%	312.5	0.01%	311	0.01%	310.5	0.01%	311.5	0.01%
	unann	3756286	85.60%	3759661	85.03%	3606570	85.05%	4252306	86.58%	3671549	85.56%
	total	4388204	100.00%	4421783	100.00%	4240659	100.00%	4911526	100.00%	4291084	100.00%
	Matched (G)	3772159	85.98%	3796935	85.88%	3654253	86.18%	4207318	85.67%	3696352	86.14%
total reads	exon_antisense	205089.5	1.74%	201559	1.70%	193870	1.64%	204364	1.73%	205197	1.74%
	exon_sense	708016.5	6.00%	696489.5	5.88%	715663.5	6.05%	642904	5.44%	696972	5.91%
	intron_antisense	268118	2.27%	252687	2.13%	258795.5	2.19%	291436	2.47%	268126.5	2.27%
	intron_sense	1198097	10.15%	1174996	9.92%	1146648	9.70%	1211955	10.25%	1159439	9.82%
	rRNA	1083576	9.18%	1336304	11.28%	1438981	12.17%	953882.5	8.07%	1250752	10.60%
	repeat	36	0.00%	35	0.00%	34.5	0.00%	37	0.00%	32.5	0.00%
	snRNA	2775.5	0.02%	3166	0.03%	2708	0.02%	2599.5	0.02%	2941	0.02%
	snoRNA	1118	0.01%	1336.5	0.01%	1009	0.01%	959.5	0.01%	1045.5	0.01%
	tRNA	191460	1.62%	202774.5	1.71%	212059.5	1.79%	122046	1.03%	147945	1.25%
	miRNA	1893986	16.04%	1815551	15.32%	1870805	15.82%	1601484	13.55%	2024702	17.15%
	unann	6252136	52.96%	6163863	52.02%	5984813	50.61%	6787436	57.43%	6045322	51.22%
	total	11804408	100.00%	11848761	100.00%	11825387	100.00%	11819102	100.00%	11802474	100.00%
	Matched (G)	10871114	92.10%	10879835	91.83%	10931188	92.44%	10838401	91.70%	10927238	92.59%

The abbreviations are defined as follows: rRNA, ribosomal RNA; repeat, repeat associated RNA; snRNA, small nuclear RNA; snoRNA, small nucleolar RNA; tRNA, transfer RNA; miRNA, microRNA; Matched (G), matched to the AD genome of *G. hirsutum*. EL, Extreme low temperature (4 °C); NL, Normal low temperature (12 °C); CK, Control (25 °C); NH, Normal high temperature (35 °C); EH, Extreme high temperature (42 °C).

**Table 2:** Summary data of degradome sequencing.

	Total					Unique				
	EL	NL	CK	NH	EH	EL	NL	CK	NH	EH
**Total tags**	10305697	9791298	10978976	9854917	12549112	3184427	2509825	2116601	1726761	2492774
**Mapping to genome**	8102865	8373876	9870519	8749166	11062310	2170434	1720751	1506369	1182370	1779768
**percentage(%)**	78.63%	85.52%	89.90%	88.78%	88.15%	68.16%	68.56%	71.17%	68.47%	71.40%

EL, Extreme low temperature (4 °C); NL, Normal low temperature (12 °C); CK, Control (25 °C); NH, Normal high temperature (35 °C); EH, Extreme high temperature (42 °C).

**Table 3:** Distribution of confirmed miRNA targets separated by category.

A	EL		NL		CK		NH		EH	
	number	percent	number	percent	number	percent	number	percent	number	percent
**Category 0**	221	37.0%	193	36.8%	191	35.8%	157	39.5%	216	41.1%
**Category 1**	45	7.5%	50	9.5%	64	12.0%	32	8.1%	36	6.8%
**Category 2**	158	26.4%	86	16.4%	79	14.8%	66	16.6%	106	20.2%
**Category 3**	30	5.0%	15	2.9%	6	1.1%	12	3.0%	20	3.8%
**Category 4**	144	24.1%	181	34.5%	193	36.2%	130	32.7%	148	28.1%
**Total**	598	100%	525	100%	533	100%	397	100%	526	100%
B										
**Category 0**	22	7.3%	28	10.6%	34	12.5%	14	7.0%	25	10.4%
**Category 1**	22	7.3%	18	6.8%	19	7.0%	18	9.0%	25	10.4%
**Category 2**	98	32.5%	84	31.9%	67	24.6%	56	28.1%	84	35.0%
**Category 3**	56	18.5%	52	19.8%	51	18.8%	28	14.1%	39	16.3%
**Category 4**	104	34.4%	81	30.8%	101	37.1%	83	41.7%	67	27.9%
**Total**	302	100%	263	100%	272	100%	199	100%	240	100%

(A) Known miRNAs; (B) Novel miRNAs. EL, Extreme low temperature (4 °C); NL, Normal low temperature (12 °C); CK, Control (25 °C); NH, Normal high temperature (35 °C); EH, Extreme high temperature (42 °C).
