# Supplementary material for: Small RNA-mediated responses to low- and high-temperature stresses in cotton
Source: Sci Rep. 2016 Oct 18;6:35558. doi: 10.1038/srep35558 (PMC5067717; doi:10.1038/srep35558)
Supplement: Supplementary Figures [file srep35558-s1.pdf]

Small RNA-mediated responses to low- and high-temperature stresses in cotton

Authors:

Qiongshan Wang, Nian Liu, Xiyan Yang, Lili Tu, Xianlong Zhang*

National Key Laboratory of Crop Genetic Improvement, Huazhong Agricultural University, Wuhan, Hubei, 430070, China

*** Corresponding author:** Xianlong Zhang, National Key Laboratory of Crop Genetic Improvement, Huazhong Agricultural University, Wuhan, Hubei, 430070, China

Telephone: +86-27-8728-0510

Fax: +86-27-8728-0196

E-mail: xlzhang@mail.hzau.edu.cn

Address: National Key Laboratory of Crop Genetic Improvement, Huazhong Agricultural University, Wuhan, Hubei 430070, China

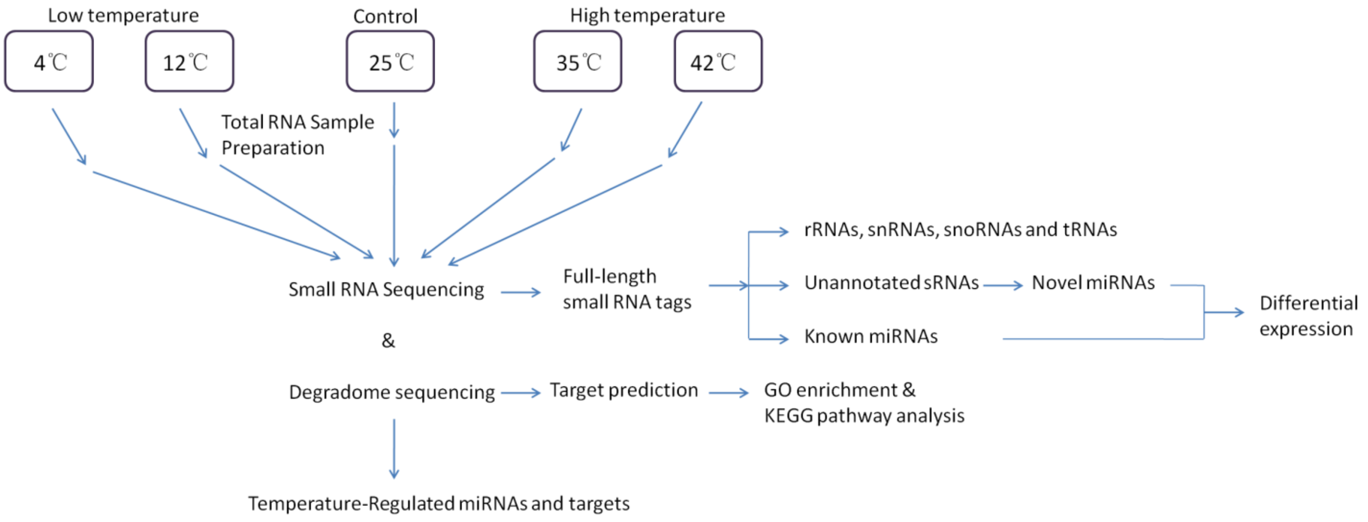

Figure S1. Flow chart of experimental strategy. Five sRNA libraries with two replicates and five mRNA degradome libraries were constructed using cotton leaves collected after different temperature treatments. The miRNAs identified from the sRNA libraries include known and novel miRNAs. The potential miRNA regulated genes were predicted based on analysis of degradome libraries.

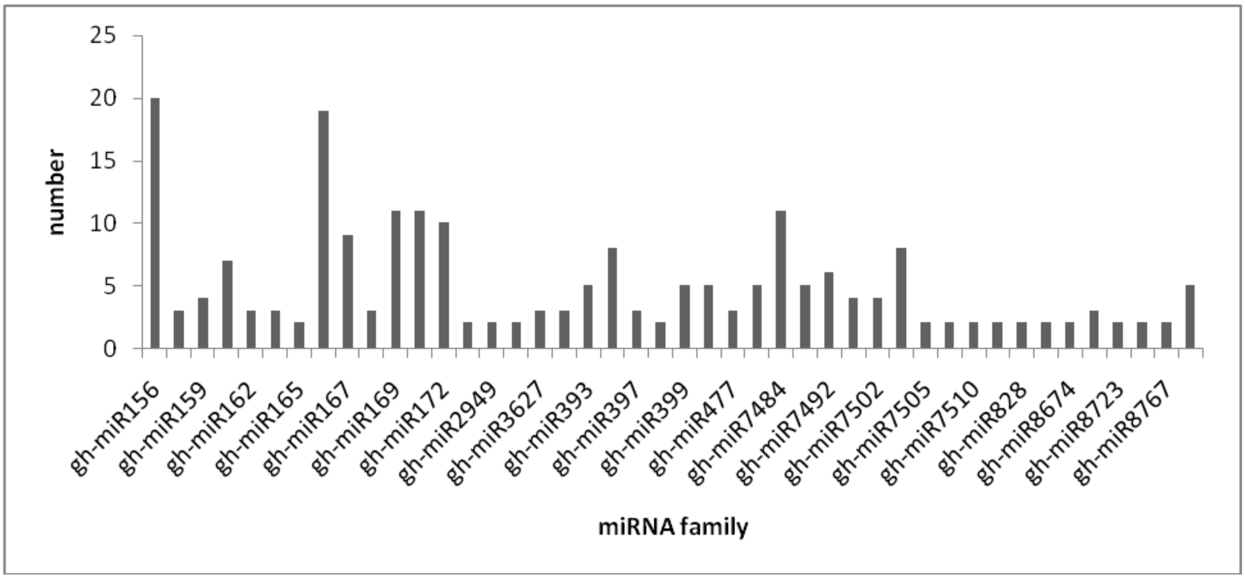

Figure S2. Member numbers of differentially expressed miRNA families.

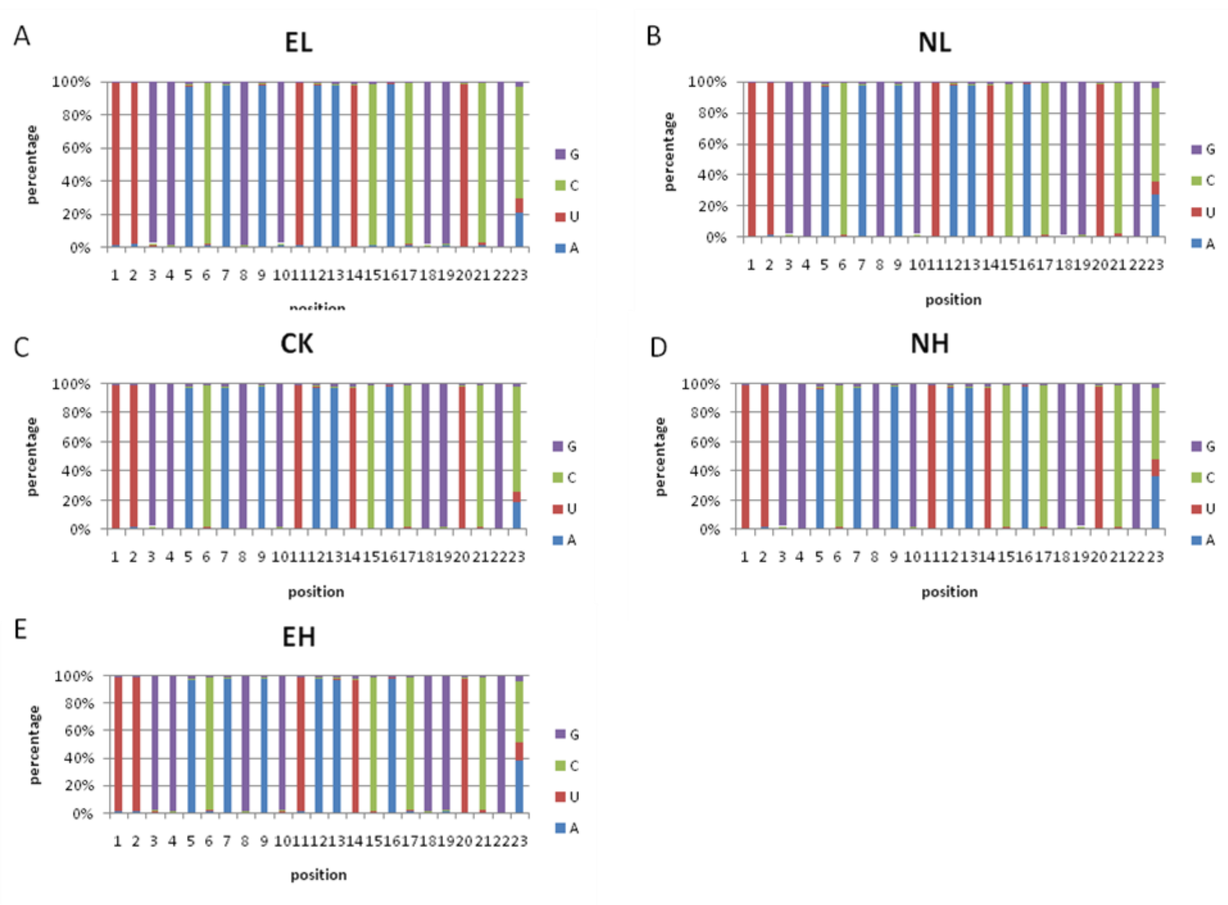

Figure S3. Nucleotide bias at each position in miRNAs among the five sRNA libraries. (A), (B), (C), (D) and (E) represent the libraries made from leaves in EL, NL, CK, NH, EH, respectively. EL, Extreme low temperature (4 ℃); NL, Normal low temperature (12 ℃); CK, Control (25 ℃); NH, Normal high temperature (35 ℃); EH, Extreme high temperature (42 ℃).

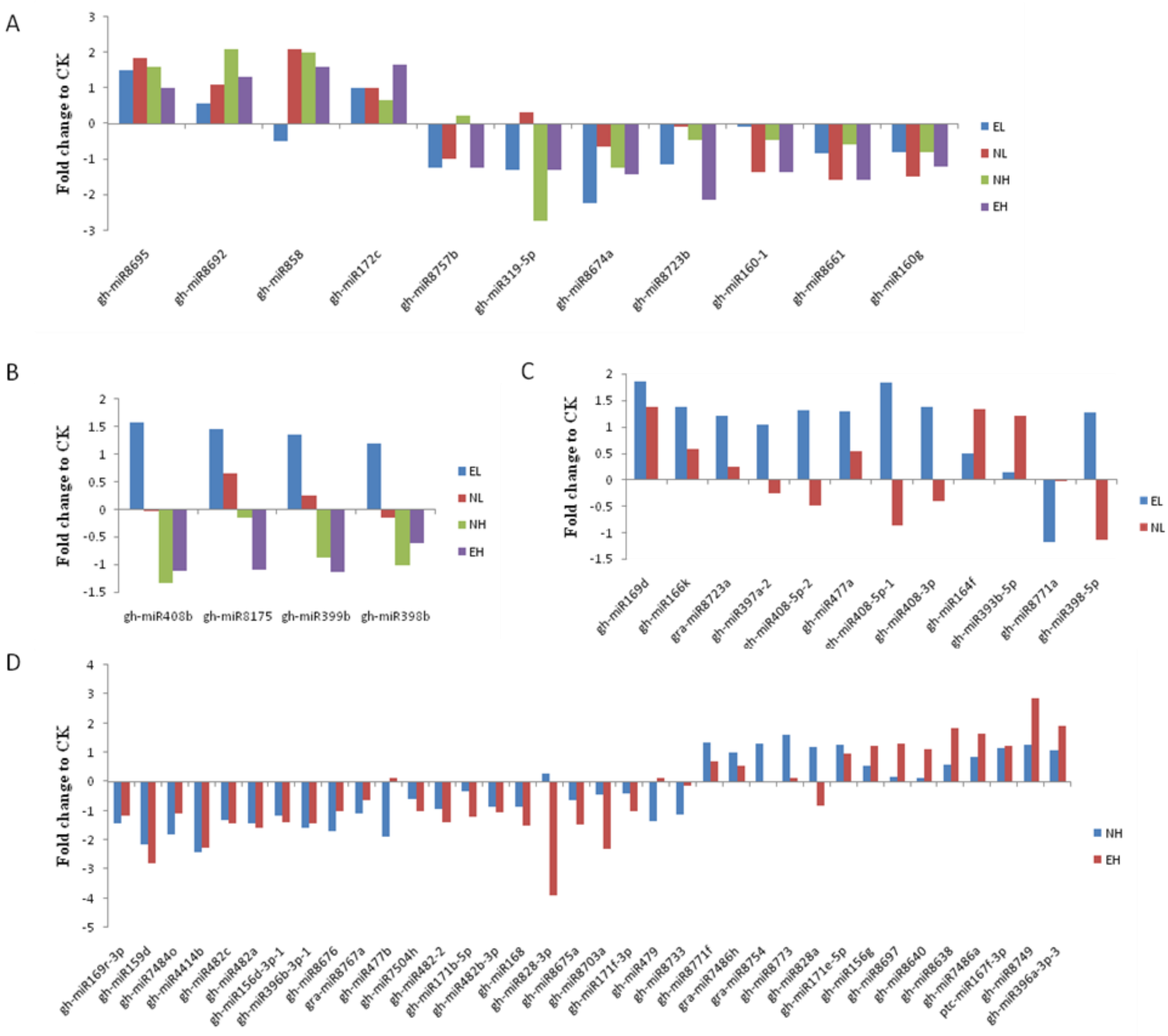

Figure S4. Comparison of known miRNA expression changes after temperature stress treatments based on deep sequencing. (A) Known miRNAs in EL, NL, NH and EH with the same expression patterns; (B) Known miRNAs in EL, NL, NH and EH with the opposite expression patterns; (C) Differently expressed known miRNAs (log2 Ratio ≥ 1 or ≤ -1) only in low temperature stress; (D) Differently expressed known miRNAs (log2 Ratio ≥ 1 or ≤-1) only in high temperature stress. EL, Extreme low temperature (4 ℃); NL, Normal low temperature (12 ℃); CK, Control (25 ℃); NH, Normal high temperature (35 ℃); EH, Extreme high temperature (42 ℃).

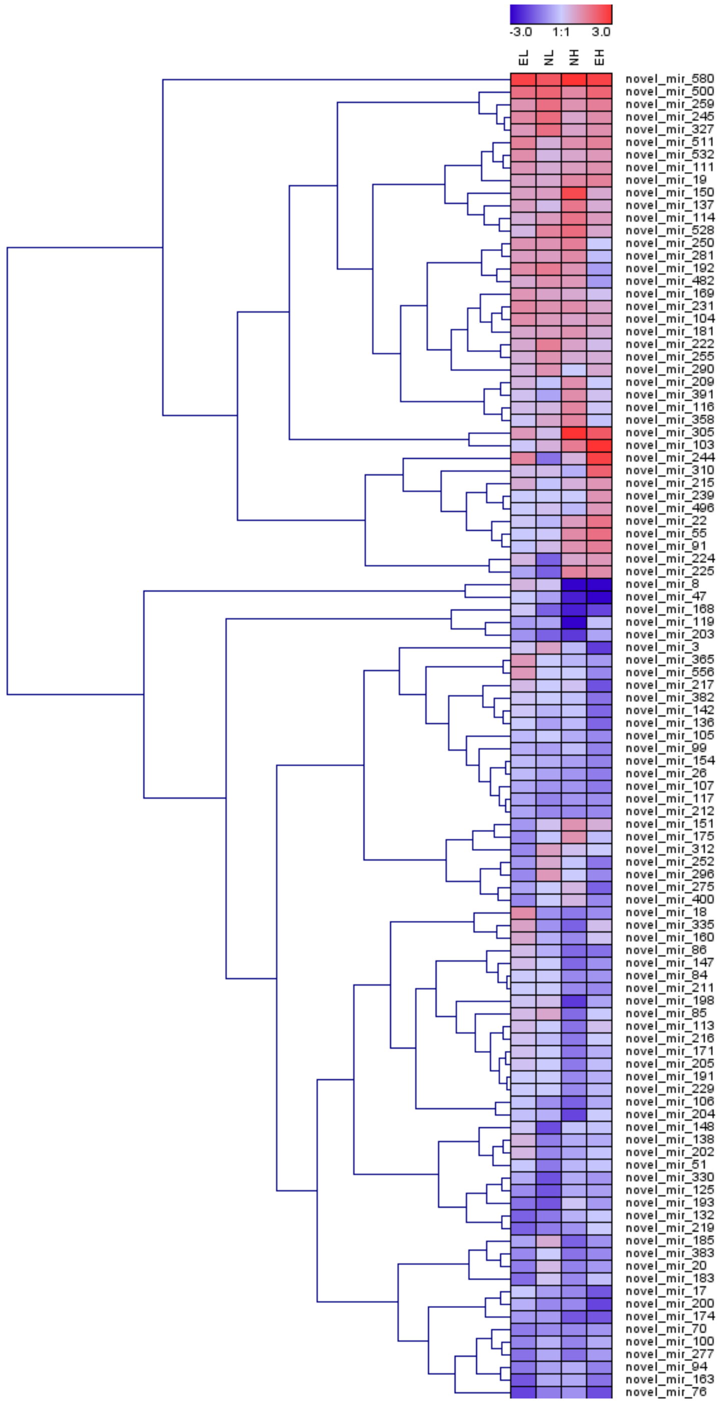

Figure S5. Heat maps of differently expressed novel miRNAs in EL, NL, NH and EH treatments. Red, up-regulated; blue, down-regulated. EL, Extreme low temperature (4 ℃); NL, Normal low temperature (12℃); NH, Normal high temperature (35 ℃); EH, Extreme high temperature (42 ℃).

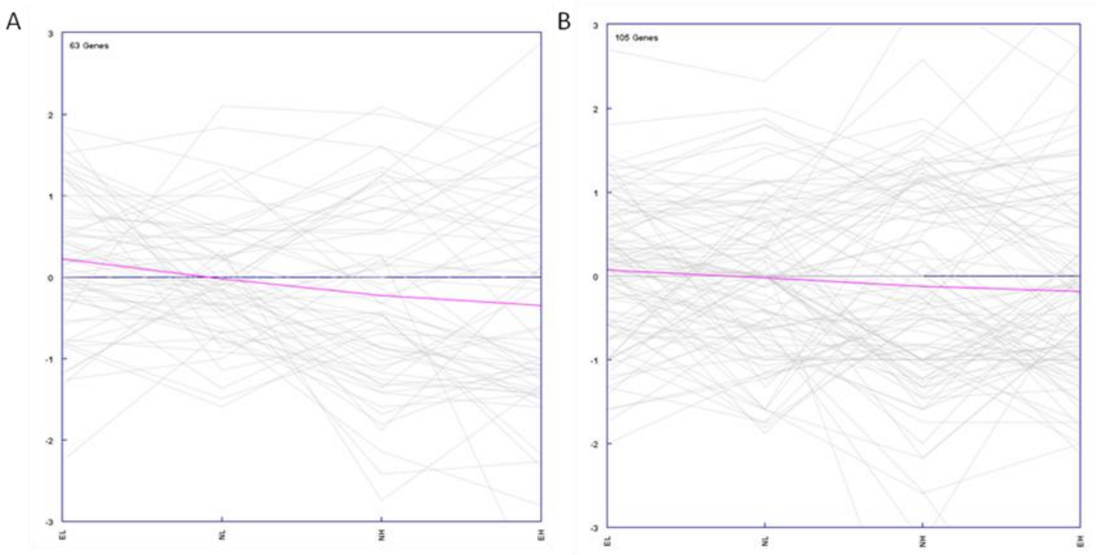

Figure S6. Cluster analysis of differentially expressed miRNAs in EL, NL, NH and EH treatments. (A) Known miRNAs, including 63 miRNAs; (B) Novel miRNAs, including 105 miRNAs. EL, Extreme low temperature (4 ℃); NL, Normal low temperature (12 ℃); NH, Normal high temperature (35 ℃); EH, Extreme high temperature (42 ℃).

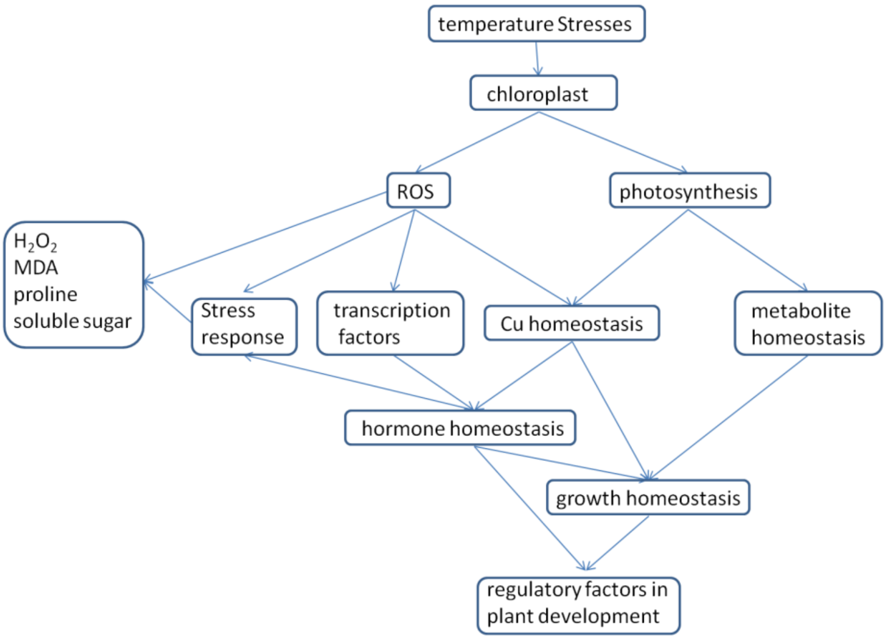

Figure S7. A potential model of small RNA-mediated regulatory network in response to temperature stresses in cotton.
